# Association between education level and cognitive trajectories from midlife to late life: the mediating role of urban–rural residence

**DOI:** 10.3389/fpubh.2026.1780451

**Published:** 2026-04-10

**Authors:** J. Xie, M. Xie, J. He, Q. Li

**Affiliations:** 1 Department of Neurology, The Affiliated Chengdu 363 Hospital of Southwest Medical University, Chengdu, Sichuan, China; 2 Mental Health Center, West China Hospital of Sichuan University, Chengdu, Sichuan, China

**Keywords:** cognitive trajectories, education level, harmonized China health and retirement longitudinal study, residence, rural area, urban area

## Abstract

**Background:**

The prevalence of cognitive impairment among older adults is rising, affecting daily living and increasing care burdens. This study explored the longitudinal relationship between education level and cognitive trajectories. And to the best of our knowledge, this study is the first to confirm the mediating role of urban–rural residence.

**Methods:**

We analyzed data from 8,117 participants aged 45 and older in the Harmonized China Health and Retirement Longitudinal Study (CHARLS, 2011–2018). Participants were grouped by education level (illiterate, elementary/middle school, high school or above) and categorized into “persistently low” or “persistently high” cognitive trajectory groups based on their average cognitive *z*-scores over time. Multivariable logistic regression was used to assess the association between education, residence, and cognitive trajectories. Sensitivity analyses and *E*-values quantified the potential influence of unmeasured confounding factors. Mediation analysis examined the role of urban–rural residence in this relationship.

**Results:**

Two cognitive trajectory patterns were identified: sustained high trajectory (57.7%) and sustained low trajectory (42.3%). Compared to illiterate participants, those with elementary or middle school education level had significantly lower risk of persistently low cognitive trajectory (Model 4: OR = 0.12, 95% CI: 0.10, 0.15). Urban residence was also independently associated with a reduced risk of persistently low cognitive trajectory (Model 4: OR = 0.60, 95% CI: 0.54, 0.66). Mediation analysis showed that urban–rural residence partially mediated the effect of education level on cognitive trajectory. Sensitivity analyses suggested the observed associations are moderately robust.

**Conclusion:**

Higher education level and urban residence are independently linked to persistently high cognitive trajectory in middle-aged and older Chinese adults, with urban–rural residence partially mediating the impact of education level. These findings highlight the enduring cognitive benefits of education level and suggest that improving living environments could enhance these effects, especially for those with lower education level. This study highlights the association between education level and cognitive trajectories in middle and old-aged adults in China. Urban–rural residence serves as a significant mediator, particularly for individuals with intermediate level of education.

## 1 Introduction

With the global population aging, the issue of cognitive impairment associated with aging has becoming increasingly severe (1). Previous study reports that the number of people worldwide living with dementia is projected to rise from 57.4 million in 2019 to 152.8 million by 2050 (2). Cognitive impairment is linked to an increased risk of functional dependence in activities of daily living (ADL) and instrumental activities of daily living (IADL), whether it occurs independently or in conjunction with acute events like stroke and chronic diseases (3). As the condition of individuals with cognitive impairment worsens, the costs of daily care and medical treatment continue to rise, placing a significant burden on healthcare systems globally (4).

Identifying modifiable factors that promote cognitive reserve and delay the onset of impairment is, therefore, a critical research priority (5). Among these factors, educational level has consistently emerged as one of the strongest predictors of lifelong cognitive health (6–9). Education demonstrated a significant effect on a wide range of Mini-Mental State Examination (MMSE) domains, encompassing orientation, attention and calculation, recall, as well as most aspects of language function (10). Individuals with higher education attainment achieved significantly higher MMSE scores (11, 12) and demonstrated better performance on complex cognitive tasks, particularly those involving executive function (13). Early education, along with other social learning experiences, can equip individuals with the skills, knowledge, and curiosity necessary to engage with intellectual challenges throughout life. As a result, cognitive function in adulthood may reflect ongoing interaction with cognitively stimulating environments (14). However, the relationship between education level and longitudinal cognitive trajectories remains incompletely understood.

It is well-established that social environmental factors play a crucial role in the onset and progression of cognitive impairment. Notably, the type of residence (urban or rural) may also influence performance on the MMSE (15). Previous studies have shown that older adults living in urban areas tend to have higher cognitive function compared to those residing in rural areas, likely due to urban residents’ greater access to education (16), occupational opportunities, healthcare resources, and social engagement in urban environments (17–19). Among individuals with mild cognitive impairment (MCI) and dementia, social isolation, stress, and physical inactivity are prevalent in both urban and rural areas (20).

As a result, the association between education level and cognitive function may evolve over time and be influenced by a range of factors, including sociodemographic, lifestyle, and health-related variables. The robustness of this association, particularly when adjusted for such confounders, requires ongoing verification through modern sensitivity analyses to better understand its impact on cognitive trajectories across the lifespan. And the specific role of urban–rural residence in mediating the relationship between education level and cognitive trajectories remains underexplored. This gap in understanding is particularly relevant given that educational attainment often intersects with access to socio-environmental resources that vary between urban and rural areas. It is therefore crucial to examine whether the benefits of education level on cognitive health are moderated by the living environment, particularly in the context of aging populations.

To address this gap, this study aims to examine the independent association between education level, urban–rural residence, and the risk of unfavorable cognitive trajectories based on the distribution of average *z*-score over time in the aging population of China. Additionally, it explores whether urban–rural residence serves as a mediating factor in the relationship between education level and cognitive trajectories.

Furthermore, this study will emphasize the transition from cross-sectional associations to longitudinal cognitive trajectories, highlighting how the trajectory-based approach using *z*-score over time adds value and distinguishes this study from prior work. By explicitly linking the study’s objectives to the life-course framework and the concept of modifiable environmental factors for cognitive protection, this research aims to offer deeper insights into cognitive protection strategies.

The findings from this study may provide valuable evidence for future strategies aimed at promoting cognitive health and protecting against cognitive decline.

## 2 Methods

### 2.1 Study population

This study was conducted based on the Harmonized China Health and Retirement Longitudinal Study (CHARLS) dataset (Version D; June 2021, Gateway to Global Aging Data), which was a prospective and nationally representative study. 17,708 participants from 450 urban and rural communities across 28 provinces in China was included by the study. A standardized face-to-face questionnaire was used to gather information of participants’ socioeconomic status, physical and psychological health, demographic characteristics, and social networks. The baseline survey of CHARLS was implemented between June 2011 and March 2012 (wave 1), with follow-up surveys conducted every 2 years. Blood samples were collected from participants in both wave 1 and wave 3, which included biomarkers such as triglyceride and glucose. The Harmonized CHARLS dataset included 25,586 total participants across Waves 1 through 4.

**Figure 1:**
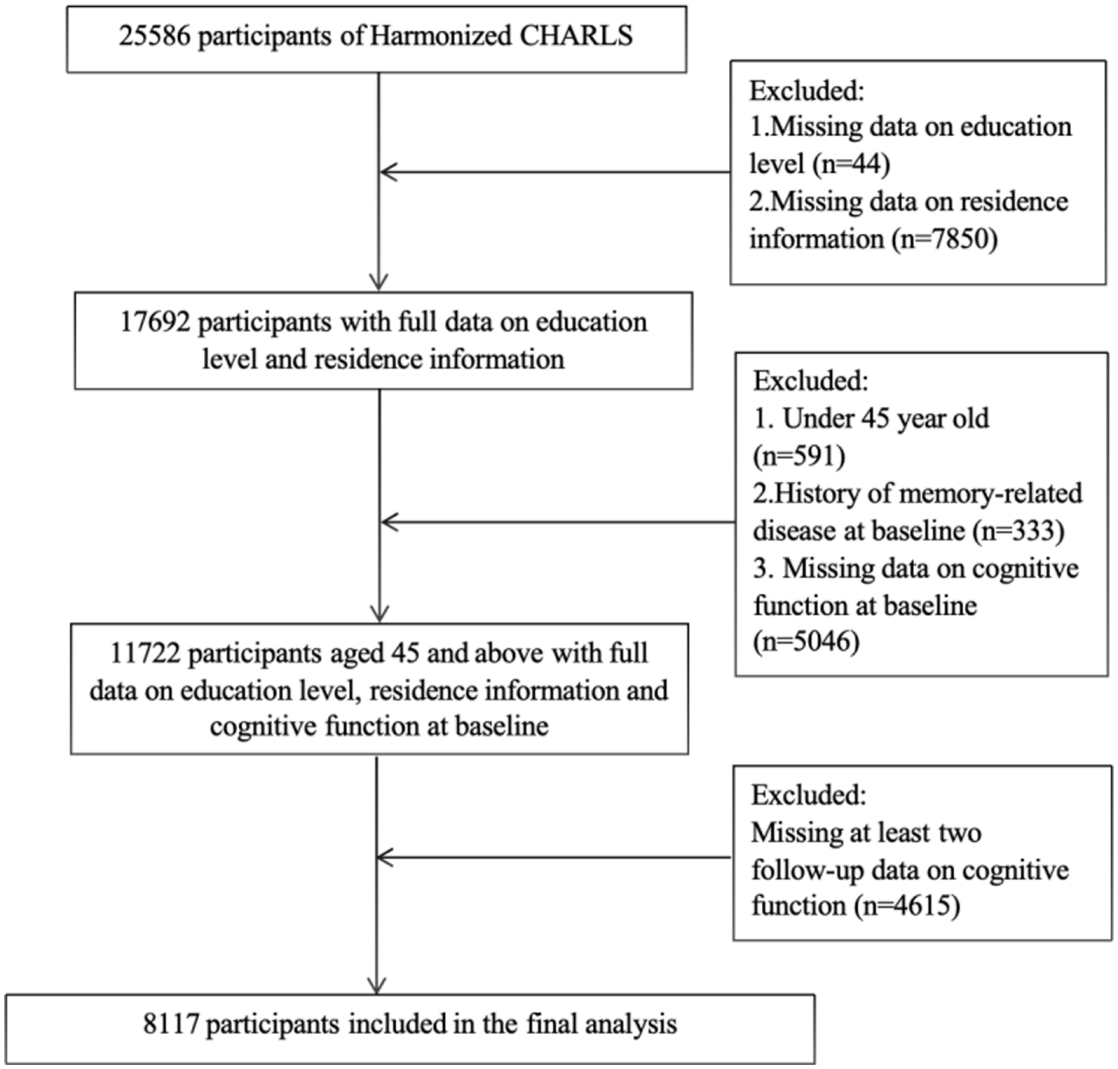
Flowchart of the participants’ selection process.

From the 25,586 participants, we excluded those who: (1) missing data on education levels (*n* = 44); (2) missing data on residence information (*n* = 7,850); (3) under 45 year old (*n* = 591); (4) history of memory-related disease at baseline (*n* = 333); (5) missing data on cognitive function at baseline (*n* = 5,046); (6) missing at least two follow-up data on cognitive function (*n* = 4,615). Finally, the statistical analysis included a total of 8,117 participants (Figure 1). The baseline data analysis of included and excluded participants is presented in Supplementary Table S1. The CHARLS study was approved by the Institutional Review Board at Peking University, all participants provided written informed consent. The study adheres to the Strengthening the Reporting of Observational Studies in Epidemiology (STROBE) guidelines (21).

### 2.2 Education level

In CHARLS, education variable was recorded as the highest level of education attained. In this study, it was harmonized into 3 categories following previous literature: illiterate, elementary or middle school, high school or above (22).

### 2.3 Residence

Residence was categorized into urban and rural according to previous research (15, 23, 24), and whether this area is rural or urban was defined by the National Bureau of Statistics of China.

### 2.4 Measurement of cognitive function

The CHARLS questionnaire assessed cognitive function with a 24-item scale derived from the Chinese MMSE (25), encompassing general ability, reaction, attention, calculation, recall, language comprehension, and self-coordination. Four cognitive dimensions were identified: orientation, computation, memory and drawing. The total score ranges from 0 to 30, with higher scores reflecting better cognitive function (26). Standardized *z*-scores of both global and the four dimensions cognitive function were calculated to enable comparisons, using the formula (value − baseline mean)/SD. In all subsequent analyses, these *z*-scores were interpreted as a proxy for cognitive function: positive values denote above-average performance, while negative values denote below-average performance relative to the population mean (6).

### 2.5 Other covariates

The following variables were included by the study due to their potential confounding effects on cognitive function: age, gender, marital status, smoking history, drinking status, society activities, depression symptom, and history of chronic disease (27–31). The Center for Epidemiologic Studies Depression Scale 10-item (CESD-10) was used to assess patients’ depressive symptoms. This 10-item scale includes both positively and reverse-scored items, each rated on a 4-point scale from 0 to 3, with higher values indicating greater frequency or intensity. The total score ranges from 0 to 30, where a higher score reflects more severe depressive symptoms. Based on previous research, a score ≥10 on the CESD-10 is considered indicative of depressive symptoms (26). Chronic disease information in CHARLS was based on self-reported physician diagnoses and initially included 14 conditions: hypertension, diabetes, liver disease, dyslipidemia, cancer, chronic lung disease, kidney disease, heart disease, stroke, gastric or digestive disorders, emotional or psychiatric problems, memory-related disease, arthritis or rheumatism, and asthma. Data on memory-related disease were excluded from the study, and a binary chronic disease variable was constructed, with individuals assigned a value of 1 if they reported having any of the remaining 13 chronic diseases, and 0 otherwise. The simplification was primarily aimed at streamlining the analysis and ensuring an adequate sample size for statistical testing. While this approach may obscure the nuanced differences in cognitive outcomes across various chronic conditions, previous studies have shown that these diseases share permanent characteristics, irreversible pathological causes, or require rehabilitation or long-term care (32). Thus, we believe that treating them as a single variable provides meaningful insights into the overall trend. Body mass index (BMI) was defined as weight in kilograms divided by the square of height in meters (kg/m^2^) (33).

### 2.6 Statistical analysis

All statistical analyses were conducted using R version 4.5.1. Continuous variables were presented as mean ± standard deviation (SD), and categorical variables as frequencies and percentages. Educational attainment was categorized as illiterate, elementary/middle school, or high school or above. Baseline characteristics were compared across education level using one-way ANOVA for continuous variables and the Chi-square test for categorical variables. *p*-values for continuous variables were adjusted for multiple comparisons using the Bonferroni method. A two-sided *p*-value <0.05 was considered statistically significant.

Cognitive performance was evaluated across four domains: memory, computation, orientation, and drawing. For the global cognitive function and four domains at each wave (2011, 2013, 2015, and 2018), a wave-specific *z*-score was calculated using the sample mean and SD of that wave. To summarize longitudinal performance, an individual’s *z*-scores across all available waves were averaged within each domain. Participants were then classified into “persistently low” or “persistently high” cognitive trajectory groups based on whether their average *z*-score fell below or above the overall sample median, respectively. This binary trajectory variable served as the primary outcome in subsequent regression analyses. This approach ensures the external validity and reproducibility of the classification criteria, thereby directly supporting our hypothesis that education level influences extreme cognitive performance. By utilizing this method, the study provides a clearer identification of key factors—such as education level and urban–rural residence—that impact cognitive advantages or disadvantages. Moreover, it minimizes the confounding effect of intermediate mixed trajectories, resulting in more intuitive and accurate estimations of effect sizes.

The relationship between education level, urban–rural residence and the odds of a persistently low cognitive trajectory was examined using multivariable logistic regression. Adjusted odds ratios (ORs) with 95% confidence intervals (CIs) were estimated. A hierarchical modeling strategy was applied to sequentially account for potential confounders using four regression models. A directed acyclic graph (DAG) was constructed to illustrate the hypothesized causal pathways and to guide the selection of confounding variables (Supplementary Figure S1).

To verify the robustness of the results, several sensitivity analyses were conducted and *E*-value was calculated to quantify the minimum association between unmeasured confounding factors with exposure and outcomes, in order to fully explain the observed associations. First, we compared the effect estimates of the minimum adjustment model 1 (age and gender only) with the comprehensive adjustment model 4. Next, stratified analysis will be conducted by grouping based on gender (male, female) and age (<60 years old, ≥60 years old). Use the third quartile to redefine the cognitive trajectory for analysis and exclude the educational years limit (educational years exceeding ±3 SD for reanalysis).

Subsequently, causal mediation analyses were performed to assess whether urban–rural residence mediated the association between education level and cognitive trajectory. We hypothesized that the relationship between the mediator and independent variables is unidirectional, meaning that the independent variable influences the mediator, which in turn affects the outcome variable. We assumed that all potential mediators (such as social activity, depression, chronic diseases, etc.) were appropriately measured and included in the analyses. We acknowledge that unmeasured confounders could impact the accuracy of causal inferences, particularly in the context of mediation effects. To address this concern, we implemented the following measures: (1) comprehensive control of known confounders; and (2) sensitivity analyses to evaluate the potential impact of unmeasured confounders on the results. Various methods were employed to assess possible biases arising from unmeasured variables, and the robustness of the findings was thoroughly verified. Analyses were stratified by education level, comparing “elementary/middle school” and “high school or above” against the “illiterate” reference group. Previous studies have indicated that memory is a key factor in cognitive impairment, and that the cognitive domains most influenced by education level are computation, reading, writing, and drawing (34). Therefore, we selected the global cognitive trajectories, along with memory and drawing trajectories for mediation analysis. The total effect (TE) was decomposed into natural direct (DE) and indirect (IE) effects. 95% CI were estimated using non-parametric bootstrapping with 1,000 replicates. The proportion mediated (PM) was calculated as IE/TE.

## 3 Results

### 3.1 Baseline characteristics

The study consisted of 8,117 participants with age of 56.9 ± 8.1 years. The baseline table reveals significant socio-demographic and clinical differences among the participants grouped by education level: 1,036 illiterate individuals, 5,793 with elementary/middle school education, and 1,288 with high school or above education (Table 1). Key finding include age, gender, residence, marital status, history of smoking and drinking, social activity, BMI, depression symptom and history of chronic diseases with *p* < 0.05. Participants with higher education level (high school or above) were younger and had lower rates of chronic diseases and depression symptoms compared to those with lower education level. Specifically, higher education level was associated with a higher proportion of urban residence, married individuals, and greater participation in social activities. In contrast, the illiterate group had a higher percentage of females, rural residence, and individuals with a history of smoking and drinking. Education also showed a positive correlation with BMI, with higher education level being linked to slightly higher BMI values. Notably, illiterate individuals reported significantly higher rates of chronic diseases and depression symptoms.

**Table 1:** Baseline characteristics of study participants by education level.

Variable	Level	Overall	Illiterate (*n* = 1036)	Elementary/middle (*n* = 5793)	High school+ (*n* = 1288)	*p*-value
Age, years		56.9 ± 8.1	59.6 ± 8.1	56.9 ± 8.1	54.9 ± 7.9	<0.001
Gender	Female	3750 (46.2)	774 (74.7)	2513 (43.4)	463 (35.9)	<0.001
	Male	4367 (53.8)	262 (25.3)	3280 (56.6)	825 (64.1)	
Residence	Rural	4741 (58.4)	769 (74.2)	3499 (60.4)	473 (36.7)	<0.001
	Urban	3376 (41.6)	267 (25.8)	2294 (39.6)	815 (63.3)	
Marital status	With spouses	7440 (91.7)	908 (87.6)	5316 (91.8)	1,216 (94.4)	<0.001
	Without spouses	677 (8.3)	128 (12.4)	477 (8.2)	72 (5.6)	
History of smoking	No	4590 (56.5)	756 (73)	3141 (54.2)	693 (53.8)	<0.001
	Yes	3527 (43.5)	280 (27)	2652 (45.8)	595 (46.2)	
History of drinking	No	4652 (57.3)	728 (70.3)	3259 (56.3)	665 (51.6)	<0.001
	Yes	3465 (42.7)	308 (29.7)	2534 (43.7)	623 (48.4)	
Social activity	No	3999 (49.3)	544 (52.5)	2949 (50.9)	506 (39.3)	<0.001
	Yes	4118 (50.7)	492 (47.5)	2844 (49.1)	782 (60.7)	
BMI, kg/m^2^		23.9 ± 4.1	23.8 ± 4.4	23.8 ± 3.9	24.6 ± 4.5	<0.001
Depression symptom	No	5572 (68.6)	605 (58.4)	3929 (67.8)	1,038 (80.6)	<0.001
	Yes	2545 (31.4)	431 (41.6)	1864 (32.2)	250 (19.4)	
Chronic diseases	No	2671 (32.9)	311 (30)	1899 (32.8)	461 (35.8)	0.012
	Yes	5446 (67.1)	725 (70)	3894 (67.2)	827 (64.2)	

The results are presented as mean ± SD, or *N* (%). Calculated by one-way ANOVA and Chi-square test. *p* < 0.05 was considered statistically significant.

### 3.2 Cognitive trajectory models

**Figure 2:**
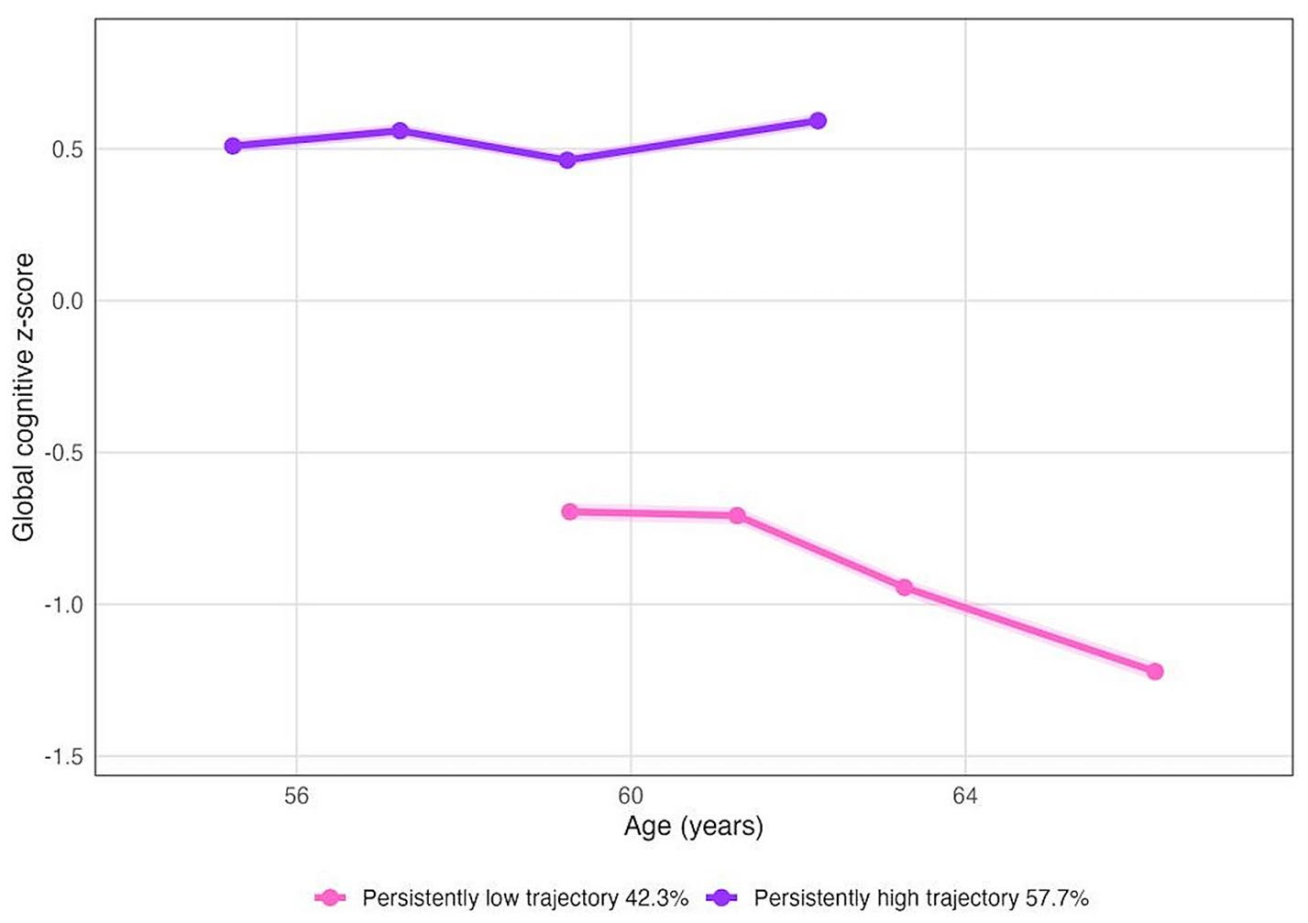
Trajectories of global cognitive *z*-score.

**Figure 3:**
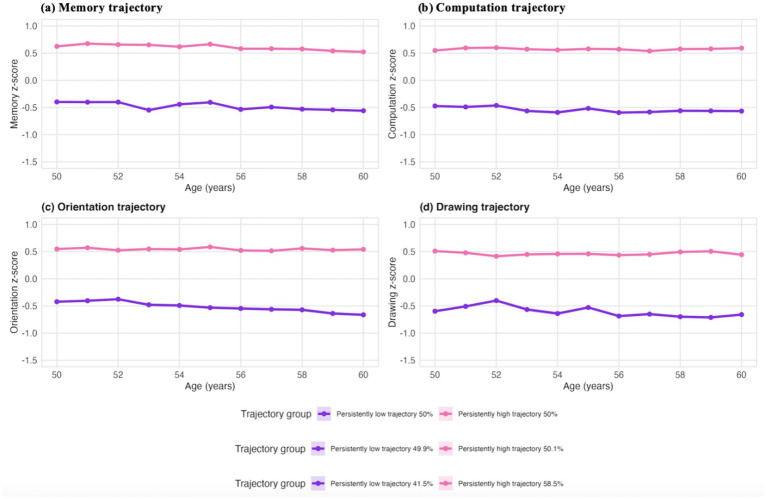
Trajectories of memory, computation, orientation, and drawing *z*-score.

Based on the age of participants at baseline and follow-up, as well as standardized global cognitive *z*-scores, we identified two longitudinal patterns of global cognitive function: persistently high trajectory (57.7%) and persistently low trajectory (42.3%) based on the distribution of average *z*-scores over time (Figure 2). And the same method was used to further plot the sustained high and low trajectory groups of four cognitive dimensions (memory, computation, orientation and drawing) (Figure 3).

### 3.3 Association between education level and urban–rural residence with cognitive trajectories

Multivariable logistic regression was employed to assess the independent associations of education level and urban–rural residence with the risk of a persistently low cognitive trajectory. Sequentially adjusted models were constructed: Model 1 adjusted for age and sex; Model 2 added sociodemographic factors (marital status and, respectively, urban–rural residence for education models or education level for urban–rural residence models); Model 3 added lifestyle factors (smoking and drinking history); Model 4 further adjusted for health status and psychosocial factors (chronic disease history, BMI, social activity, and depression symptoms).

#### 3.3.1 Association of education level with cognitive trajectories

Higher education level was strongly and consistently associated with a lower risk of persistently low cognitive trajectories across all cognitive domains (Table 2). For global cognition, compared to illiterate participants, those with elementary/middle school education had significantly lower odds of cognitive decline (Model 4: OR = 0.12, 95% CI: 0.10, 0.15), while the protection was even more pronounced for those with high school or above education level (Model 4, OR = 0.03, 95% CI: 0.03, 0.04). This inverse dose–response relationship was consistent for specific domains: orientation (Model 4: OR = 0.22, 95% CI: 0.18, 0.26 for elementary/middle; OR = 0.10, 95% CI: 0.08, 0.12 for high school or above), memory (Model 4: OR = 0.22, 95% CI: 0.18, 0.26 and OR = 0.07, 95% CI: 0.06, 0.09, respectively), computation (Model 4: OR = 0.26, 95% CI: 0.22, 0.31 and OR = 0.13, 95% CI: 0.10, 0.16, respectively), and drawing (Model 4: OR = 0.19, 95% CI: 0.16, 0.22 and OR = 0.07, 95% CI: 0.06, 0.09, respectively). All associations remained stable across the adjustment models.

**Table 2:** Logistic regression models of education level and cognitive trajectories.

Models	Education exposure	Proportion in persistently low trajectory (*n*/*N*)	Model 1 OR (95% CI)	Model 2 OR (95% CI)	Model 3 OR (95% CI)	Model 4 OR (95% CI)
Global cognitive trajectories	Illiterate	933/1036	Ref.	Ref.	Ref.	Ref.
	Elementary/middle	2900/5793	0.12 (0.09, 0.14)	0.13 (0.10, 0.16)	0.13 (0.10, 0.16)	0.12 (0.10, 0.15)
	High school+	225/1288	0.03 (0.02, 0.03)	0.03 (0.02, 0.04)	0.03 (0.02, 0.04)	0.03 (0.03, 0.04)
Orientation trajectories	Illiterate	865/1036	Ref.	Ref.	Ref.	Ref.
	Elementary/Middle	2852/5793	0.19 (0.16, 0.23)	0.22 (0.18, 0.26)	0.22 (0.18, 0.26)	0.22 (0.18, 0.26)
	High school+	317/1288	0.07 (0.05, 0.08)	0.09 (0.07, 0.11)	0.09 (0.07, 0.11)	0.10 (0.08, 0.12)
Memory trajectories	Illiterate	860/1036	Ref.	Ref.	Ref.	Ref.
	Elementary/Middle	2917/5793	0.20 (0.17, 0.24)	0.22 (0.18, 0.26)	0.22 (0.18, 0.26)	0.22 (0.18, 0.26)
	High school+	279/1288	0.06 (0.05, 0.07)	0.07 (0.05, 0.09)	0.07 (0.05, 0.09)	0.07 (0.06, 0.09)
Computation trajectories	Illiterate	846/1036	Ref.	Ref.	Ref.	Ref.
	Elementary/Middle	2837/5793	0.25 (0.21, 0.29)	0.26 (0.22, 0.30)	0.26 (0.22, 0.30)	0.26 (0.22, 0.31)
	High school+	371/1288	0.11 (0.09, 0.13)	0.12 (0.10, 0.15)	0.12 (0.10, 0.15)	0.13 (0.10, 0.16)
Drawing trajectories	Illiterate	856/1036	Ref.	Ref.	Ref.	Ref.
	Elementary/Middle	2383/5793	0.17 (0.15, 0.21)	0.18 (0.16, 0.22)	0.19 (0.16, 0.22)	0.19 (0.16, 0.22)
	High school+	235/1288	0.06 (0.05, 0.07)	0.07 (0.06, 0.09)	0.07 (0.06, 0.09)	0.07 (0.06, 0.09)

Model 1: age and sex adjusted. Model 2: residence and marital status were added to Model 1. Model 3: smoking and drinking history were added to Model 2. Model 4: chronic disease history, BMI, social activity and depression symptom were added to Model 3.

#### 3.3.2 Association of urban–rural residence with cognitive trajectories

Urban residence was independently associated with a lower risk of low cognitive trajectories, though the magnitude of this effect was more moderate compared to education level and attenuated after full adjustment (Table 3). In Model 1, urban residence showed significantly lower odds for global cognitive decline (OR = 0.42, 95% CI: 0.38, 0.46). After adjusting for education level and other covariates in Model 4, the protective effect remained significant but attenuated (OR = 0.60, 95% CI: 0.54, 0.66). A similar attenuation was observed in specific cognitive domains: orientation (Model 1: OR = 0.33, 95% CI: 0.30, 0.36; Model 4: OR = 0.44, 95% CI: 0.39, 0.48), memory (Model 1: OR = 0.48, 95% CI: 0.44, 0.53; Model 4: OR = 0.67, 95% CI: 0.60, 0.74), computation (Model 1: OR = 0.65, 95% CI: 0.59, 0.71; Model 4: OR = 0.85, 95% CI: 0.77, 0.94), and drawing (Model 1: OR = 0.53, 95% CI: 0.48, 0.58; Model 4: OR = 0.71, 95% CI: 0.64, 0.78).

**Table 3:** Logistic regression models of urban–rural residence and cognitive trajectories.

Models	Residence exposure	Proportion in persistently low trajectory (*n*/*N*)	Model 1 OR (95% CI)	Model 2 OR (95% CI)	Model 3 OR (95% CI)	Model 4 OR (95% CI)
Global cognitive trajectories	Rural	2742/4741	Ref.	Ref.	Ref.	Ref.
	Urban	1316/3376	0.42 (0.38, 0.46)	0.54 (0.49, 0.60)	0.55 (0.49, 0.60)	0.60 (0.54, 0.66)
Orientation trajectories	Rural	2860/4741	Ref.	Ref.	Ref.	Ref.
	Urban	1174/3376	0.33 (0.30, 0.36)	0.40 (0.36, 0.44)	0.40 (0.37, 0.44)	0.44 (0.39, 0.48)
Memory trajectories	Rural	2679/4741	Ref.	Ref.	Ref.	Ref.
	Urban	1377/3376	0.48 (0.44, 0.53)	0.61 (0.55, 0.68)	0.61 (0.56, 0.68)	0.67 (0.60, 0.74)
Computation trajectories	Rural	2547/4741	Ref.	Ref.	Ref.	Ref.
	Urban	1507/3376	0.65 (0.59, 0.71)	0.80 (0.73, 0.88)	0.81 (0.73, 0.89)	0.85 (0.77, 0.94)
Drawing trajectories	Rural	2285/4741	Ref.	Ref.	Ref.	Ref.
	Urban	1189/3376	0.53 (0.48, 0.58)	0.68 (0.61, 0.75)	0.68 (0.62, 0.75)	0.71 (0.64, 0.78)

Model 1: age and sex adjusted. Model 2: education level and marital status were added to Model 1. Model 3: smoking and drinking history were added to Model 2. Model 4: Chronic disease history, BMI, social activity and depression symptom were added to Model 3.

### 3.4 Sensitivity analyses

**Figure 4:**
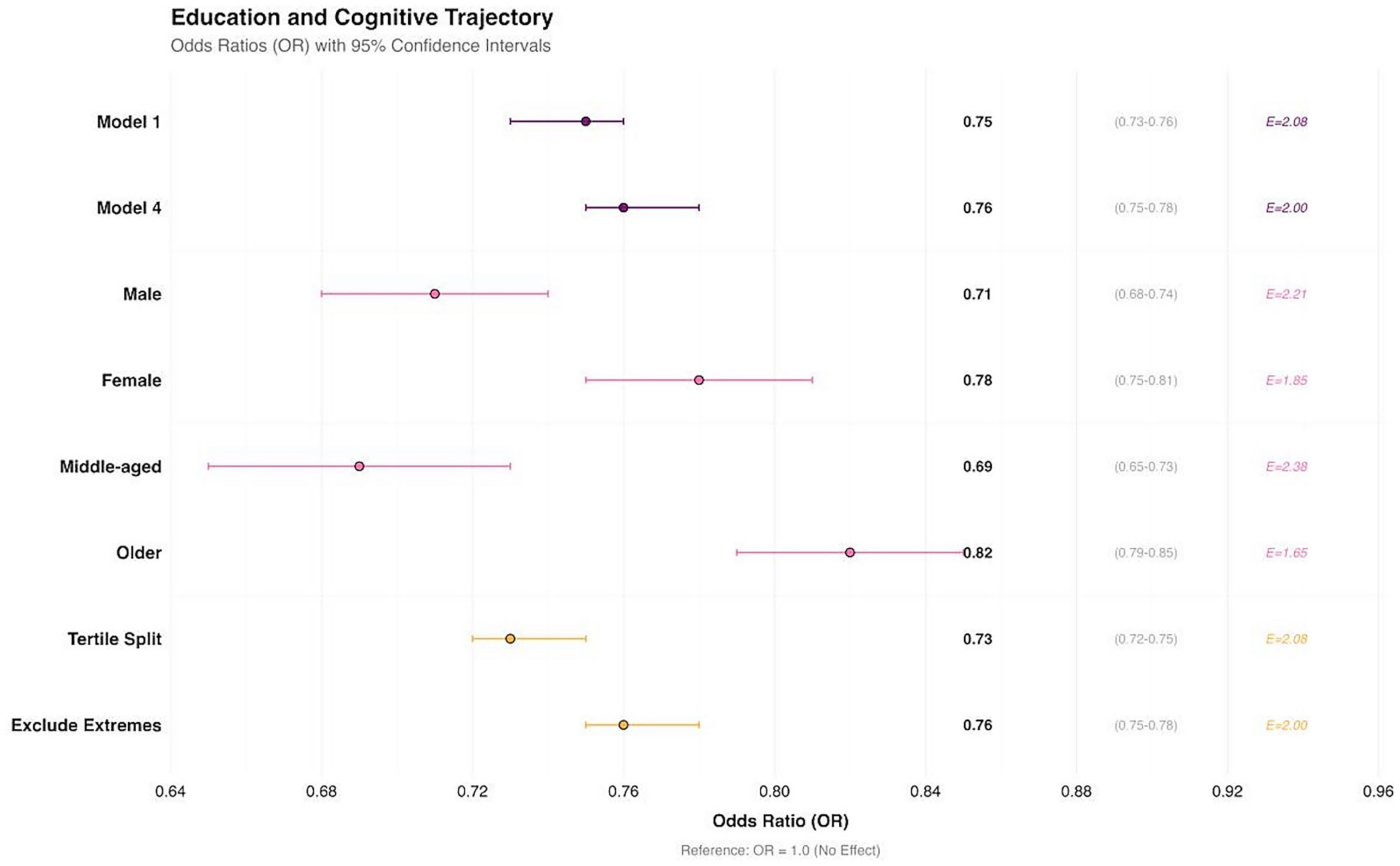
Sensitivity analyses of education level and cognitive trajectory. Model 1: age and sex adjusted. Model 4: age, sex, urban–rural residence, marital status, history of smoking and drinking, history of chronic disease, BMI, social activity, and depression symptom adjusted.

To evaluate the robustness of the association, we conducted a series of sensitivity analyses (Figure 4). Detailed results of these sensitivity analyses are presented in Supplementary Table S2. The association remained consistent when comparing the minimally adjusted model (Model 1, adjusted only for age and sex: OR = 0.75, 95% CI: 0.73–0.76) with the fully adjusted model (Model 4: OR = 0.76, 95% CI: 0.75–0.78).

In stratified analyses, a protective association was observed in both males (OR = 0.71, 95% CI: 0.68–0.74) and females (OR = 0.78, 95% CI: 0.75–0.81), as well as in middle-aged (OR = 0.69, 95% CI: 0.65–0.73) and older participants (OR = 0.82, 95% CI: 0.79–0.85). When educational years were analyzed by tertiles, the effect estimate remained similar (OR = 0.73, 95% CI: 0.72–0.75). Furthermore, after excluding individuals with extreme educational years, the association persisted (OR = 0.76, 95% CI: 0.75–0.78).

We calculated *E*-values to assess the potential influence of unmeasured confounding. The *E*-value for the primary association was 2.08, suggesting that an unmeasured confounder would need to be associated with both education level and cognitive trajectory by risk ratios of at least 2.08-fold each to fully explain away the observed association. For key sensitivity analyses, *E*-values ranged from 1.65 to 2.38, indicating that the observed associations are moderately robust to potential unmeasured confounders.

### 3.5 Urban–rural residence mediated the association between education level and cognitive trajectories

**Figure 5:**
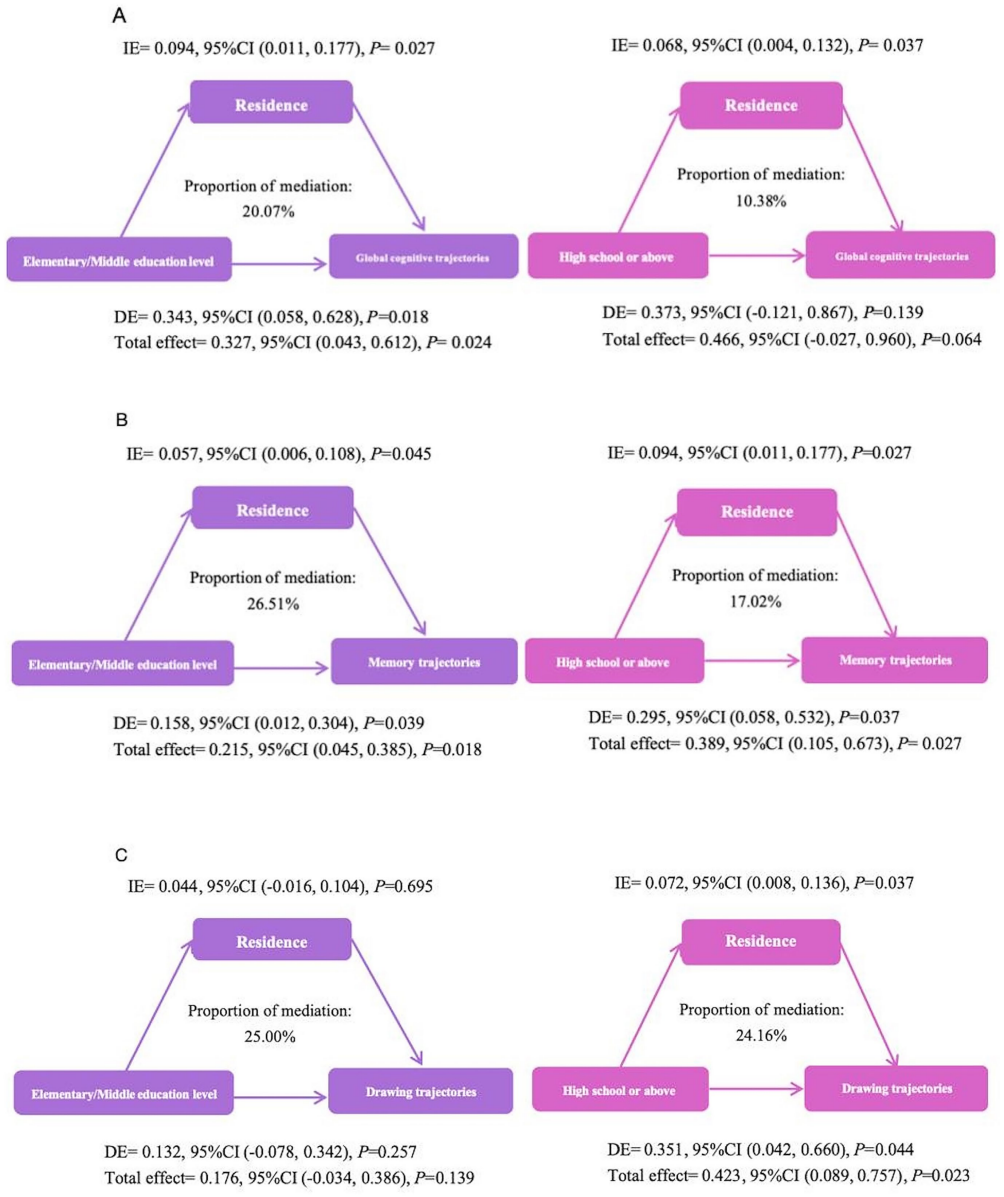
Mediation analysis of urban–rural residence on the interaction between education level and global **(A)**, memory **(B)**, and drawing **(C)** cognitive trajectories.

A mediation analysis was conducted to examine whether urban–rural residence mediated the association between education level and cognitive trajectories. The total effect (TE), direct effect (DE), and indirect effect (IE) through urban–rural residence are summarized below (Figure 5).

#### 3.5.1 Global cognitive trajectories

For individuals with an elementary/middle school education, urban–rural residence significantly mediated the association with a lower risk of persistently low global cognitive trajectory (Figure 5A). The IE was 0.094 (95% CI: 0.011, 0.177; *p* = 0.027), accounting for 20.07% of the TE. The DE of education remained significant (DE = 0.373, 95% CI: 0.058, 0.688; *p* = 0.018).

#### 3.5.2 Memory trajectories

Urban–rural residence significantly mediated the association between education level and memory trajectories for both education level groups (Figure 5B). Among the elementary/middle school group, the IE was 0.057 (95% CI: 0.006, 0.108; *p* = 0.045), accounting for 26.51% of the TE. The DE was also significant (DE = 0.158, 95% CI: 0.012, 0.304; *p* = 0.039).

For the high school or above group, the IE was 0.094 (95% CI: 0.011, 0.177; *p* = 0.027), explaining 17.02% of the TE. The DE remained significant (DE = 0.295, 95% CI: 0.058, 0.532; *p* = 0.037).

#### 3.5.3 Drawing trajectories

For drawing trajectories, a significant mediation effect was observed only in the higher education group (Figure 5C). Among individuals with a high school or above education level, urban–rural residence significantly mediated the relationship (IE = 0.072, 95% CI: 0.008, 0.136; *p* = 0.037), explaining 24.16% of the TE. Both the DE (DE = 0.351, 95% CI: 0.042, 0.660; *p* = 0.044) and TE (TE = 0.423, 95% CI: 0.089, 0.757; *p* = 0.023) were statistically significant.

## 4 Discussion

We included 8,117 nationally representative middle and old-aged Chinese adults from CHARLS to examine the association between education level and cognitive trajectories across global and domain-specific cognitive functions based on averaged *z*-scores over 7 years. This large-scale longitudinal study provides robust evidence of a strong, graded association between higher educational level and a lower risk of persistently low cognitive trajectories, corroborating previous findings (35). The association was robust to comprehensive adjustment for sociodemographic, lifestyle, and health-related confounders, exhibited consistency across key subgroups, and showed moderate resilience to potential unmeasured confounding. Furthermore, our mediation analysis offers novel evidence that urban residence partially explain the cognitive benefits of education level, particularly for individuals with elementary/middle school education level and in specific cognitive domains like memory.

Our primary finding of a potent, dose–response relationship between education level and cognitive trajectories preservation aligns robustly with the cognitive reserve hypothesis. The concept of cognitive reserve suggests that the brain actively attempts to respond to pathological changes through existing cognitive processing methods or compensation mechanisms. And utilize sustained cellular, molecular, and systemic level repair and plasticity for preservation and maintenance. Both reserve and maintenance may be influenced by genetic and environmental factors, such as education or exercise (36–38). Therefore, individuals with higher education level may have more efficient or high-capacity neural networks and stronger adaptability when faced with age-related or disease-related changes (38). The strikingly low odds ratios in our study, especially for the high school or above group (OR = 0.03 for global cognition), underscore education level as one of the most powerful modifiable factors identified for lifelong cognitive trajectories in this population. The consistency of this pattern across four cognitive domains (orientation, memory, computation, and drawing) suggests that education level confers a broad-based neuroprotective benefit rather than enhancing only specific, education-related skills. However, the effect sizes may also indicate residual confounding or measurement issues. In particular, factors such as socio-economic status, early life experiences, and access to healthcare may contribute to these observed associations. Therefore, further studies are needed to confirm the robustness of these findings.

We found an independent association between urban residence and low cognitive trajectory protection, aligning with previous study (24, 39, 40), which may reflect the advantages of environmental and social structural resources. A study from the United States shows that individuals who live in rural areas at any point in their lives, as well as those who move from rural areas to cities from childhood to adulthood, have poorer cognitive trajectories in later life compared to those who have always lived in urban areas. This may be due to the limited resources (like libraries, schools), healthcare services, and other health facilities that support the development of health cognition in rural areas, leading to lifelong differences in cognitive health (41). Cognitive stimulation can improve global cognitive function, memory, orientation and praxis in older adults (4). Urban environments typically offer greater access to cognitively stimulating activities, superior healthcare services, and richer social networks (42). The attenuation of the residence effect after adjusting for education level and other covariates in our study (e.g., OR for global cognition shifted from 0.42 to 0.60) indicates that a portion of the urban advantage is explained by the higher educational attainment of urban residents and correlated lifestyle factors. This is consistent with the findings of previous research (24).

Our mediation analysis reveals the complex mode of action of place of residence in the education cognition relationship. The significant mediation role of urban–rural residence, accounting for 20.07% of the TE in the group of elementary/middle school education level for global cognition trajectories, suggests that for individuals with moderate education level, moving to or residing in an urban area may be an important mechanism that translates educational capital into better cognitive trajectories. This could be due to urban environments providing the opportunities and infrastructure necessary to actualize the skills and potentials developed through education attainment. Previous studies have shown that individuals with high lifelong career achievement have a lower risk of cognitive impairment compared to individuals with low career achievement (38). Participants living in urban area may also reduce the risk of cognitive trajectories by gaining more career opportunities. We found that the mediation effects were diminished or non-significant among individuals with high school or above education level, consistent with prior research (24). A plausible interpretation is that higher education level provides such substantial cognitive reserve and access to resources that the additional contextual advantages of urban living become relatively less important. It is worth noting that the IE of urban–rural residence on the drawing trajectory of participants with elementary/middle school education level did not reach a significant level (IE = 0.044, 95% CI: −0.016, 0.104). This finding may suggest that for the population with basic education level, their drawing ability (which may rely more on basic visual spatial functions) is influenced differently by the environmental resources related to urban–rural residence than by areas that rely more on advanced cognitive processing or acquired knowledge (such as memory). In addition, in the group of high school or above education level, although the IE of residence on the global cognitive trajectory is significant, its’ DE and TE becomes insignificant. This may suggest that for the participants with higher education level, the protective effect of education level is so strong that the additional contribution of the living environment becomes relatively vague statistically, or its pathway of action is replaced or masked by other unmeasured factors such as occupational complexity or social network quality. In these cases, the lack of a substantial TE does not necessarily diminish the importance of the IE. Instead, it underscores the importance of considering the more complex pathways through which the environment may influence cognitive trajectories. The mediation effect here is not meant to imply a dominant or exclusive role of urban–rural residence, but rather to highlight its potential, albeit modest, contribution to the observed cognitive trajectories within this subgroup. These results suggest that the relationship between education level and urban–rural residence is multifaceted, and it should not be viewed as a simple one-way mediation pathway. The inconsistency in the observed effects between education groups emphasizes the need to consider various environmental and sociocultural factors that could modify the relationship between education level and cognitive trajectories. Further research is needed with larger sample sizes to better detect these more subtle effects and to explore other potential mediators, such as occupational complexity or the quality of social networks. Nonetheless, the E-values ranged from 1.65 to 2.38 in our sensitivity analyses. These results further indicate that the findings are moderately robust to potential unmeasured confounding.

Our findings have significant public health implications. They underscore investment in education level as a foundational strategy for promoting lifelong cognitive trajectories at the population level. For middle and old-aged adults with limited education level, our study highlights that enhancing environmental enrichment—perhaps through community-based cognitive stimulation, improved access to services, and social engagement in rural areas, could be a promising compensatory intervention strategy.

While our study offers valuable insights into cognitive aging, there are several limitations to consider. First, the use of average *z*-scores to categorize cognitive trajectories provides a general overview, but may not fully capture individual variations in cognitive changes. Future research could benefit from more advanced techniques, such as latent class analysis (LCA) or growth mixture modeling (GMM), to identify subgroups with distinct cognitive trajectories. Second, we did not incorporate early-life factors, such as childhood cognitive ability, parental education, and early socioeconomic environment, which are known to influence later-life cognitive function. Including these factors in future studies would enhance our understanding of the developmental roots of cognitive aging. Third, the binary classification of urban–rural residence may oversimplify the complex role of living environments on cognitive trajectories. A more nuanced approach, considering varying degrees of urbanization and access to resources, could provide a clearer picture of how these factors shape cognitive aging.

Despite these limitations, our sensitivity analysis supports the robustness of our findings. The associations between education level, urban–rural residence, and cognitive trajectories remained consistent across different model specifications, providing a strong foundation for future research to further explore these relationships.

## 5 Conclusion

In conclusion, this study highlights the association between education level and cognitive trajectories based on averaged *z*-scores over time in middle and old-aged adults in China. Urban–rural residence serves as a significant mediator, particularly for individuals with intermediate level of education. These findings not only emphasize the long-term cognitive benefits of education level, but also offer new insights into how living environments influence the translation of educational advantages into healthy cognitive aging. By enhancing our understanding of how socio-environmental factors, such as education level and urban–rural residence, interact to shape cognitive outcomes in aging populations, this study contributes to the literature on cognitive aging. Our findings suggest that improving educational opportunities, especially for disadvantaged groups, and optimizing urban environments may serve as effective strategies to promote cognitive health in aging populations.

## Data Availability

The original contributions presented in the study are included in the article/Supplementary material, further inquiries can be directed to the corresponding author.
