# Supplementary material for: Association between education level and cognitive trajectories from midlife to late life: the mediating role of urban–rural residence
Source: Front Public Health. 2026 Apr 10;14:1780451. doi: 10.3389/fpubh.2026.1780451 (PMC13106350; doi:10.3389/fpubh.2026.1780451)
Supplement: Supplementary file 1 [file Data_Sheet_1.docx]

**Table S1: The Demographic Characteristics of Included and Excluded Participants (Mean(SD)/N(%))**

| **Characteristic** | **Excluded Sample**  **N = 17,434^1^** | **Included Sample**  **N = 8,117^1^** | **p-value^2^** |
| --- | --- | --- | --- |
| Age (Years) | 59.8 (11.5) | 56.9 (8.1) | <0.001 |
| Unknown | 8,041 | 0 |  |
| BMI (Body Mass Index) (kg/m2) | 24.0 (40.8) | 24.3 (30.6) | <0.001 |
| Unknown | 10,930 | 1,018 |  |
| CESD Score | 9.3 (6.6) | 7.5 (5.9) | <0.001 |
| Unknown | 9,549 | 0 |  |
| Education level |  |  | <0.001 |
| Illiterate | 5,214 (30%) | 1,036 (13%) |  |
| Elementary or middle school | 10,229 (59%) | 5,793 (71%) |  |
| High school or above | 1,947 (11%) | 1,288 (16%) |  |
| Unknown | 44 | 0 |  |
| Gender |  |  | <0.001 |
| Female | 9,476 (54%) | 3,750 (46%) |  |
| Male | 7,950 (46%) | 4,367 (54%) |  |
| Unknown | 8 | 0 |  |
| Marital status |  |  | <0.001 |
| With spouses | 7,978 (84%) | 7,440 (92%) |  |
| Without spouses | 1,566 (16%) | 677 (8.3%) |  |
| Unknown | 7,890 | 0 |  |
| Residence |  |  | 0.005 |
| Rural | 5,779 (60%) | 4,741 (58%) |  |
| Urban | 3,777 (40%) | 3,376 (42%) |  |
| Unknown | 7,878 | 0 |  |
| Smoking | 3,390 (36%) | 3,527 (43%) | <0.001 |
| Unknown | 8,026 | 1 |  |
| Drinking | 3,343 (36%) | 3,463 (43%) | <0.001 |
| Unknown | 8,041 | 4 |  |
| Social activity | 3,460 (43%) | 4,117 (51%) | <0.001 |
| Unknown | 9,361 | 1 |  |
| Chronic disease | 6,419 (69%) | 5,428 (67%) | 0.025 |
| Unknown | 8,131 | 65 |  |
| Depression | 3,383 (43%) | 2,545 (31%) | <0.001 |
| Unknown | 9,549 | 0 |  |
| *^1^*Mean (SD); N (%) | | | |
| *^2^*Wilcoxon rank sum test; Pearson's Chi-squared test | | | |

**Figure S1. Directed acyclic graph (DAG) depicting the hypothesized causal relationships between education, residence and cognitive function**


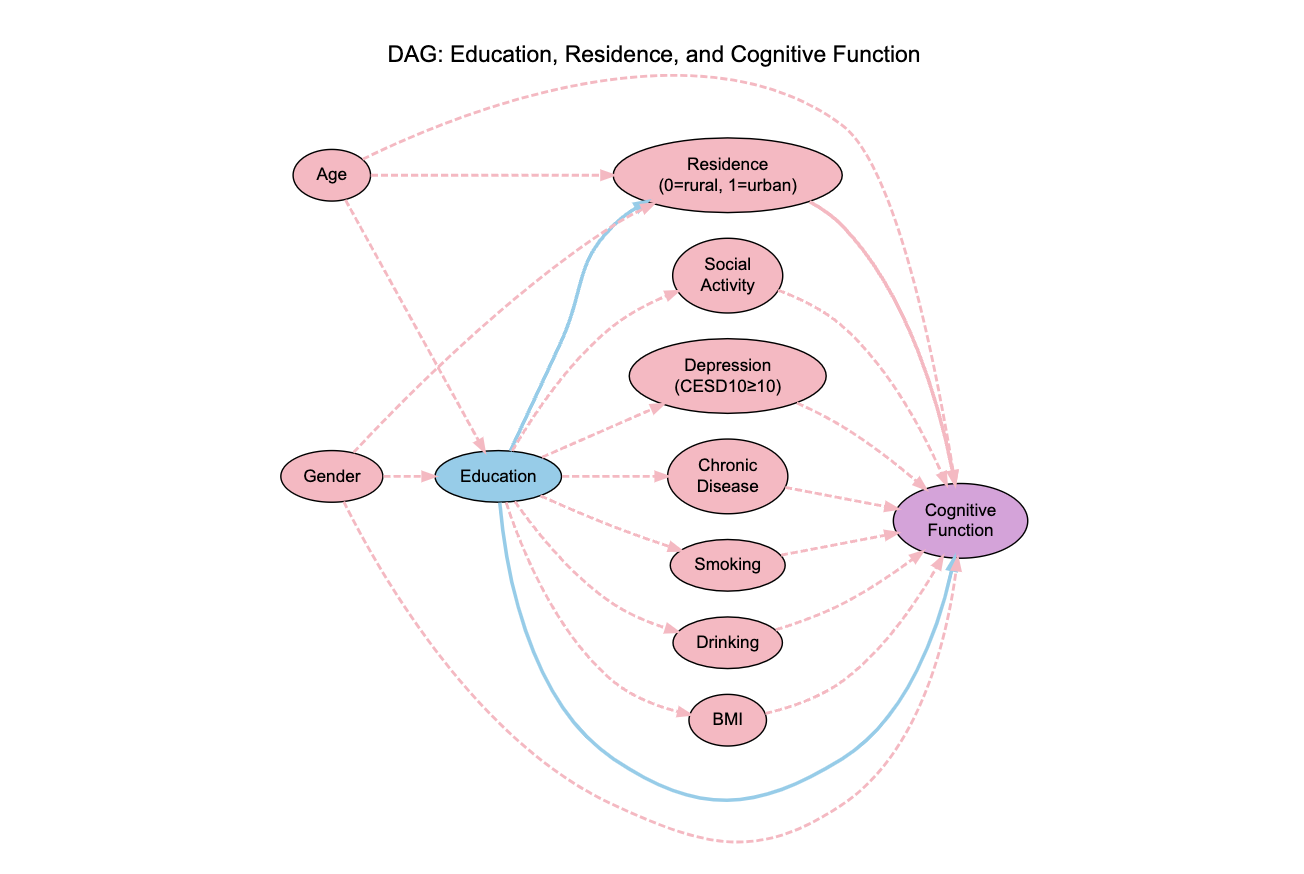


**Table S2: Sensitivity analyses of the association between education level and low cognitive trajectories**

| **Analysis Type** | **OR** | **95%CI** | **E-value** | **Risk Reduction** |
| --- | --- | --- | --- | --- |
| Model 1(Adjusted for age and sex) | 0.75 | 0.73-0.76 | 2.08 | 25.0% |
| Model 4(Fully adjusted) | 0.76 | 0.75-0.78 | 2.00 | 24.0% |
| Male | 0.71 | 0.68-0.74 | 2.21 | 29.0% |
| Female | 0.78 | 0.75-0.81 | 1.85 | 22.0% |
| Middle-aged (<60 years) | 0.69 | 0.65-0.73 | 2.38 | 31.0% |
| Older (≥60 years) | 0.82 | 0.79-0.85 | 1.65 | 18.0% |
| Alternative outcome definition: Tertile Split | 0.73 | 0.72-0.75 | 2.08 | 27.0% |
| Excluding participants with extreme education years | 0.76 | 0.75-0.78 | 2.00 | 24.0% |
